# Unusual Presentation of Cutaneous B-Cell Lymphoma Atop an Arteriovenous Fistula Made for Renal Dialysis

**DOI:** 10.5826/dpc.1102a05

**Published:** 2021-03-08

**Authors:** Ahmed Abdelbary, Hadir Shakshouk

**Affiliations:** 1 Department of Dermatology, Andrology and Venerology, Alexandria University, Egypt

**Keywords:** cutaneous B cell lymphoma, skin malignancy, dermatopathology, lymphoma

## Introduction

Primary cutaneous B-cell lymphomas (PCBCLs) are uncommon diseases with heterogeneous clinical pictures and prognoses. PCBCLs are classified into 3 main subtypes: cutaneous marginal zone lymphoma (PCMZL), cutaneous follicle center lymphoma (PCFCL), and cutaneous diffuse large B-cell lymphoma, leg-type (DLBCL-LT). Each subtype has a distinct clinical presentation and histopathological, molecular and immunologic profiles. They also differ considerably in prognosis and treatment [1]. We report an atypical case of PCBCL, leg type (LT) arising atop an arteriovenous (AV) fistula made for renal dialysis in a young woman.

## Case Presentation

**Figure 1:**
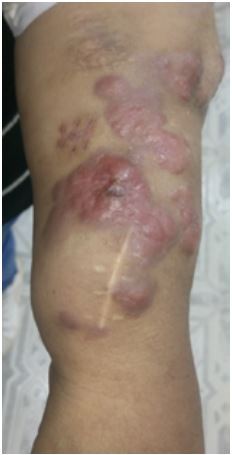
Multiple erythematous papules and nodules on the forearm, one showing superficial ulceration.

A 32-year-old woman presented with multiple tender erythematous papules of 1 month’s duration on her right forearm on top of an AV fistula that was made for renal dialysis. She was a known renal failure patient since her early twenties and had been on renal dialysis since then. She reported that lesions on her forearm were increasing in number and size and becoming more painful. Physical examination revealed multiple erythematous papules and nodules on the forearm. They were firm in consistency and tender on palpation. One of the nodules showed superficial ulceration (Figure 1). Given the nodule arose on top of an AV fistula, our clinical differential diagnosis included a wide range of vascular tumors.

**Figure 2:**
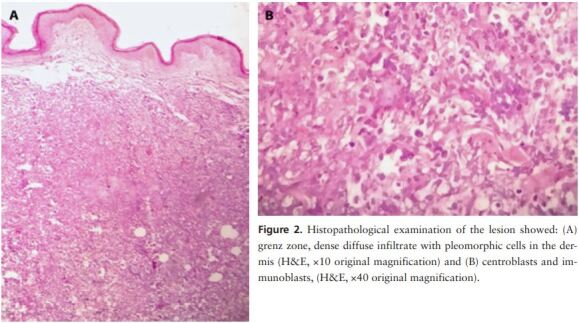
Histopathological examination of the lesion showed: (A) grenz zone, dense diffuse infiltrate with pleomorphic cells in the dermis (H&E, ×10 original magnification) and (B) centroblasts and immunoblasts, (H&E, ×40 original magnification).

**Figure 3:**
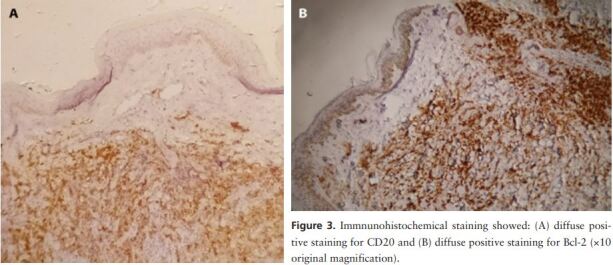
Immnunohistochemical staining showed: (A) diffuse positive staining for CD20 and (B) diffuse positive staining for Bcl-2 (×10 original magnification).

Histopathological examination revealed grenz zone and diffuse infiltration of large pleomorphic cells occupying the whole dermis (Figure 2). Some mitotic figures were noted. CD20 was found to be diffusely positive for all these atypical cells and CD4 was negative. This highlighted the possibility of cutaneous B-cell lymphoma. Further immunohistochemical analysis showed diffuse Bcl-2 positive staining and few scattered Bcl-6 positive staining (Figure 3). CD10 and MUM1 were negative. A battery of laboratory investigations and radiological imaging were unremarkable. Given these histopathological and immunohistochemical findings, a diagnosis of primary cutaneous B-cell lymphoma, leg type was made. The patient received one cycle of R-CHOP (rituximab, doxorubicin, vincristine, cyclophosamide and prednisone), but she unfortunately died.

## Conclusion

PCBCL-LT is considered an aggressive type of cutaneous B-cell lymphoma. It presents clinically as nodules of violaceous color with frequent ulceration. It is predominant in elderly females. Secondary involvement of other organs can occur shortly after diagnosis that leads to a poor prognosis. Distinction for systemic lymphoma can be difficult [1]. Typical histopathological features include a dense diffuse infiltrate composed of centroblasts and immunoblasts occupying the dermis and extending to the hypodermis. Immunohistochemical staining shows positivity for the usual B cell markers, CD20 and CD-79a, and characteristically diffuse positivity for Bcl-2 and MUM1. Bcl-2 positivity has been linked to poor prognosis in some studies [2]. MUM1 has been shown to be negative in PCBCL-LT in some reports. Given its poor prognosis, PCBCL-LT requires aggressive treatment. Chemotherapy with R-CHOP is considered the first line [2].

Primary cutaneous B-cell lymphomas are rare tumors and often represent a diagnostic challenge. Extensive immunohistochemical studies are crucial to determining the type in addition to laboratory and radiological workups to exclude extracutaneous involvement.
